# “Men can take part”: examining men’s role in supporting self-injectable contraception in southern Malawi, a qualitative exploration

**DOI:** 10.1186/s12978-022-01476-w

**Published:** 2022-08-09

**Authors:** Lucy W. Ruderman, Catherine Packer, Akuzike Zingani, Philemon Moses, Holly M. Burke

**Affiliations:** 1 Reproductive, Maternal, Newborn and Child Health Division, FHI 360, 359 Blackwell Street, Suite 200, Durham, NC 27701 USA; 2 Centre for Health, Agriculture Development Research and Consulting (CHAD), Blantyre, Malawi

**Keywords:** DMPA-SC, Family planning, Contraceptive injectables, Self-injection, Self-care, Male Engagement Framework, Qualitative analysis, Partner communication, Malawi, Sub-saharan Africa

## Abstract

**Background:**

The male engagement framework for reproductive health, which presents men as family planning users, supportive partners, and agents of change, is being increasingly incorporated into family planning strategies worldwide. We applied this framework to understand the perspectives of and role that men play in supporting the use of self-injection of subcutaneous depot medroxyprogesterone acetate (DMPA-SC).

**Methods:**

We conducted a qualitative analysis using data from a study conducted in southern Malawi to develop and test a counseling message to introduce DMPA-SC and self-injection. We conducted 4 focus group discussions (FGD) with male community leaders and partners of DMPA-SC users, 13 interviews and FGDs with public and private sector family planning providers, and 30 interviews with female clients. We explored all participant groups’ perspectives on what could facilitate or prevent women from choosing self-injection, including views on men’s attitudes towards DMPA-SC and self-injection.

**Results:**

Overall, participants expressed ways that men could be engaged as cooperative users, supportive partners, and agents of change, and felt that this would help build a more supportive environment for DMPA-SC self-injection use. Men held favorable opinions of DMPA-SC self-injection: they felt that it is useful, described ways they could actively and emotionally support their partners in its use, and described their role in normalizing it.

**Conclusions:**

We suggest that DMPA-SC self-injection has the potential to be both a female-controlled and a cooperative method, based on the ability for women to use it autonomously and the option to encourage male partner involvement (only where the woman welcomes this). Shifting the conversation from viewing men as a barrier to men as a resource may allow us to harness the social capital of men and transform traditional power dynamics, therefore establishing more enabling environments to support autonomy and choice for DMPA-SC and self-injection use.

## Plain language summary

Self-injectable contraception (DMPA-SC) has the potential to expand family planning access. Once users are trained to self-inject, they can obtain multiple units and self-inject every three months privately, without needing to return to a health provider. Considering men’s role in supporting self-injection can inform family planning programs’ male engagement strategies.

We conducted a study in Southern Malawi which included interviews and focus group discussions with male community leaders and partners of DMPA-SC users, family planning providers, and female family planning clients. We applied the male engagement framework to these data to understand the potential roles men can play as cooperative users, supportive partners, and agents of change. We found that men can support their partners in DMPA-SC self-injection use through actively participating in the injection process, providing emotional support and encouragement, and advocating for other men and communities to accept self-injection and family planning use.

We acknowledge that encouraging male participation could potentially lead to women’s autonomy being restricted, so offer concrete suggestions to create an enabling environment that keeps women’s and girls’ needs central. For example, we propose that program materials expand their description of DMPA-SC self-injection to include a potentially cooperative option, which may be appropriate for women who want to involve their partners. Further, we suggest that social and behavioral change programs channel men’s social capital in order to normalize self-injection. When their traditional role as norm influencers is harnessed, men may encourage their communities to support women’s autonomy and choice for DMPA-SC and self-injection use.

## Background

Engaging men in family planning can improve reproductive health and gender equality outcomes [1–3]. The male engagement framework for involving men in reproductive health, which presents men as family planning users, supportive partners, and agents of change [4], is increasingly being incorporated into family planning strategies worldwide [5, 6]. While recognizing the multiple roles men can play to improve reproductive health outcomes for men and women, the framework also acknowledges the importance of protecting and encouraging women’s agency [7]. In this paper, we apply the male engagement framework to qualitative data collected during a study that developed and tested a counseling message to introduce subcutaneous depot medroxyprogesterone acetate (DMPA-SC).

Injectables are the most popular contraceptive method in sub-Saharan Africa [8]. DMPA-SC, available in an all-in-one prefilled, auto-disabled Uniject™ injection system known as Sayana® Press, has been found to be easy to use and suitable for administration by lower-cadre health workers and women themselves [9–11]. Recent research has shown that women who self-injected DMPA-SC had significantly higher rates of continuation than those receiving provider-injected DMPA-SC, demonstrating its role as a self-care innovation [12, 13]. Self-injection requires fewer clinic visits and, therefore, users may save time and money and may inject themselves in a private location at their convenience. Published literature has also described self-injection as particularly promising for reaching younger women, new users of contraceptive methods, and covert users [14, 15]. Based on the feasibility and acceptability of DMPA-SC self-injection, Malawi’s Ministry of Health (MOH) introduced DMPA-SC (both provider-administered and self-injected) country wide to expand the contraceptive method mix [16].

However, partner acceptability has been identified as having important implications for uptake and continuation of self-injection [17]. For example, a study in Uganda found that the primary reason for discontinuing DMPA-SC self-injection was husband’s disapproval [18]. Despite evidence linking partner approval to uptake and continuation, research has yet to explore the role men play in DMPA-SC and self-injection beyond general community acceptability [19, 20]. Furthermore, while understanding men’s perspectives is pivotal to overcoming the barriers contraception-users face in accessing family planning [21, 22], such perspectives are seldom reflected in the literature. To address this gap, we applied the male engagement framework to qualitative data from men, family planning providers and female clients to identify ways men already are or could be engaged to support DMPA-SC use, particularly self-injection, as well as men’s perspectives on the method. We also offer recommendations for how the family planning sector can engage men as self-injection scales up globally.

### Application of the male engagement framework

We applied the male engagement framework—specifically, involving men as family planning users, supportive partners, and agents of change—to our qualitative data to guide our thinking on how to involve men in the uptake and use of DMPA-SC and self-injection among their female partners. Here we describe the framework’s components and how we defined them in the context of DMPA-SC self-injection.

#### Men as family planning users

Family planning methods have been categorized into: female-controlled, male-controlled, and cooperative contraceptive methods [7]. Traditionally, men are seen as family planning users when they actively use male-controlled methods such as external condoms or vasectomy or cooperative methods which require participation and use by both partners such as the standard days method. Injectable contraceptives, including self-injection, are traditionally viewed as female-controlled. However, we explored self-injection as a cooperative option, whereby males could be encouraged to “use” the method alongside their female partner(s) in cases where male involvement is desired—not to serve as gatekeepers to family planning.

#### Men as supportive partners

The framework’s construct of men as supportive partners is defined as men who both improve their own knowledge and attitudes toward family planning and have a positive impact on their partner’s method choice and usage. Their support not only reflects positive couple communication and joint decision-making, but also sensitivity toward their partners’ interest in effectively using voluntary contraception, including DMPA-SC self-injection in the case of our analysis. Men helping alleviate their partners’ fears by comforting them during self-injection would be an example of being a supportive partner.

#### Men as agents of change

In representing men as potential agents of change in family planning practices, the framework recognizes, and suggests channeling, the social capital and status of men within a given community to help diminish barriers to contraception use. In a recent application of the framework, authors described how taking advantage of gender differences may be viewed as gender-exploitative or gender-accommodating. However, critically examining and harnessing the power of men to take action against harmful gender norms and barriers to family planning may instead be gender-transformative [7]. In our analysis, we considered men’s support of self-injection as gender-transformative in the sense that use of their power to endorse self-injection could contribute to community change toward greater autonomy for women.

## Methods

### Design and overview

We conducted a study in southern Malawi whose primary objective was to develop and test a counseling message to introduce DMPA-SC and self-injection. Further information on the methodology, sampling study facilities, and primary results from that study are reported elsewhere [23]. During the formative phase of the study, we collected qualitative data from male community leaders and partners of DMPA-SC users, as well as private and public sector family planning providers and female clients, to develop and refine the counseling message.

A secondary objective of the study was to explore men’s attitudes and perspectives on DMPA-SC and self-injection. To meet this objective, we asked all participant groups about their perspectives on what could facilitate or prevent women from choosing to self-inject, including their views on men’s attitudes towards DMPA-SC and self-injection. This paper reports findings from a qualitative analysis of data collected from those men, family planning providers, and female clients.

### Sample selection and recruitment

We conducted 4 focus group discussions (FGDs) with men in Mangochi and Thyolo districts, 13 interviews and FGDs with private and public sector family planning providers in the city of Blantyre and in Mangochi and Thyolo districts, and 30 interviews with female clients from the same service delivery channels. FGDs aimed to include 6 to 10 participants each. Sample sizes were based on previous research, which showed that conducting 6 interviews is sufficient to develop overarching themes and useful interpretations in qualitative data [24] and that 80% saturation of study themes can be reached with 3 FGDs and 90% within 5 FGDs [25].

To sample public sector providers, we clustered study facilities by geographic zone in Mangochi and Thyolo districts based on the feasibility and logistics of data collection. We randomly selected 4 zones per district, from which we randomly selected 2 zones to include facility-based providers and 2 to include community-based health surveillance assistants (HSAs). We randomly sorted de-identified lists of public-sector providers from each facility in the 4 zones and selected 1 to 3 providers from each one, for a total of up to 12 providers selected per list, according to their availability. We identified and invited all eligible private sector providers from 3 pharmacies in Blantyre, and 1 private clinic in Mangochi and 1 in Thyolo. Due to the COVID-19 pandemic, we expanded our data collection to offer providers the option of participating in an FGD or a one-on-one in-person or phone interview. Eligibility criteria were being 18 or older, having experience providing DMPA-SC and self-injection, and being willing to be audio recorded during the interview/FGD.

A similar method was used to select 4 zones in each district from which female clients and male partners of DMPA-SC users and community leaders would be invited to participate. These zones were not necessarily the same or different than the zones selected for the providers. To recruit female clients, we randomly selected one facility per zone and, for each facility, randomly selected one facility-based provider and one HSA to request a de-identified list of eligible clients from the previous 1 to 3 months. We then randomly selected one client from each list. We worked with the private sector providers to recruit clients in a similar way. Clients were eligible if they were ages 18–49, had never used DMPA-SC, and had sought family planning services at the specific service delivery channel.

For the men’s FGDs, we worked directly with a subset of the selected HSAs who had provided the lists of aforementioned eligible female clients. Four HSAs generated lists of potential FGD participants, with the aim of recruiting half community leaders and half partners of DMPA-SC users, while noting that some community leaders were also partners of DMPA-SC users. HSAs were instructed to identify community leaders who were considered “influential,” such as religious leaders, or those who provided a social good, such as teachers. Eligibility criteria included being 18 or older, being identified as an influential person, or being a married partner of a DMPA-SC user (provider- or self-injected), and willing to be audio recorded during the FGD.

### Data collection

Data were collected in 2 steps. First, we conducted FGDs and interviews with providers in July 2020 and analyzed the transcripts. We then conducted interviews and FGDs with female clients and male partners and community leaders from September to October 2020. The study team recruited women and men from Malawi with prior experience collecting data for family planning research studies and trained them on research ethics and qualitative interviewing skills. These trained qualitative facilitators, including AZ, used semi-structured interview and FGD guides, respective to each participant group, which explored topics such as what participants knew about DMPA-SC and self-injection, personal- and community-level views on the methods, why they believed women would or would not choose to self-inject, and how they could support or be supported in DMPA-SC self-injection use (men and female clients). Interview and FGD guides were developed based on several years of experience conducting quantitative and qualitative research about DMPA-SC self-injection in Mangochi district in Malawi [12, 15, 26–29].

After providing informed consent, participants completed a short demographic survey using KoBoToolbox software [30] to collect age and sex, as well as answered questions as applicable about experience using contraception (female clients and men) and experience providing family planning services (providers), including counseling on DMPA-SC and self-injection. The team of facilitators conducted interviews and FGDs primarily in Chichewa, with some use of English for certain terminology, audio recorded them (with permission from participants), and transcribed, and translated verbatim into English transcripts.

### Data analysis

We summarized the participant demographic survey data descriptively using Excel. The transcript data were summarized in 3 participant group-specific rapid analysis matrices in Excel [31, 32] based on the relevant guides and preliminary review of transcripts. The study Co-Investigators (CP and HMB) and two qualitative analysts (LWR and KR, mentioned in Acknowledgements) analyzed the transcript data. CP and KR completed the provider and female client matrices, with consistency ensured by each analyst independently filling out one matrix per participant type and discussing any discrepancies. HMB filled out the matrix for the 4 men’s FGDs, and CP summarized the main themes for each participant group into detailed memos using the matrices.

Additional analysis consisted of LWR reviewing the men’s FGD matrix and, using a data-driven approach, identifying and summarizing themes related to men’s perspectives into a detailed memo. Then LWR conducted a targeted review of the female client and provider matrices and memos to identify data related to men’s perspectives and added this into a new comprehensive memo. In preliminary analysis, there did not appear to be differences by district or sector (private vs. public), therefore, we do not report by these factors.

Once the comprehensive memo was complete, LWR categorized the themes according to the male engagement framework. “Men as Users” included themes related to direct participation in the self-injection process or men taking an active approach to help their partners successfully self-inject. Themes related to men providing emotional support and acceptance of self-injection and their reasons for doing so were categorized under “Men as Supportive Partners.” “Men as Agents of Change” included themes related to men channeling their acceptance of self-injection into influencing norms within a community or society. In addition, the framework’s recent application suggests that engaging men in social and behavioral change (SBC) efforts can improve family planning knowledge and normalize its use [7]. SBC is crosscutting for all 3 constructs of the framework and can contribute to creating a more enabling environment for family planning use. In our analysis, we identified themes related to expanding general knowledge and attitudes around DMPA-SC self-injection and family planning and categorized them as “Engaging men in SBC”.

### Ethical considerations

This study was reviewed and approved by Malawi’s National Health Sciences Research Committee, FHI 360’s Protection of Human Subjects Committee, and Marie Stope’s International’s Ethics Review Committee. All research participants signed informed consent forms prior to participating in an FGD or interview.

### Reflexivity

As part of the qualitative research process, researchers and analysts must reflect on their positionality and inherent biases in order to conduct meaningful research [33]. To ensure our work remained culturally grounded and responsive, the team from FHI 360, based in the US, worked closely with the Malawi Centre for Health, Agriculture Development Research and Consulting (CHAD) to design and implement this study. This team has worked together on previous research about DMPA-SC self-injection in Malawi, and closely collaborated with the Malawi MOH. Colleagues from the Malawi MOH suggested incorporating men’s FGDs into this study to understand men’s perceptions of DMPA-SC and self-injection. Several CHAD staff had pivotal roles in the data collection, quality assurance, analysis, and authorship of this paper. The US- and Malawi-based teams met regularly online throughout the study, which allowed for continuous and open discussion about data collection, analysis and interpretation to ensure that the data were represented as accurately and objectively as possible.

## Results

In total, 37 men participated in 4 FGDs in Mangochi and Thyolo. In Blantyre, Mangochi, and Thyolo, 64 providers (58 from public sector and 6 from private sector) participated in 9 FGDs and 4 interviews, and 30 female clients (12 from public and 18 from private sector) participated in interviews.

Participants of FGDs with men consisted of approximately half community leaders and half partners of DMPA-SC users. Their average age was 39 years, all had at least one child, and 38% had completed secondary school (Table 1). Almost 95% of the men’s partners had previously used the intramuscular version of DMPA (DMPA-IM), 62% had self-injected DMPA-SC, and 38% had ever received provider-administered DMPA-SC. For nearly half the men’s partners (49%), the most recently used family planning method was DMPA-SC self-injection.

**Table 1:** Sociodemographic characteristics of community leaders and partners of DMPA-SC users (FGDs with men)

Characteristic	Total (N = 37) N (%)
*District*	
Mangochi	20
Thyolo	17
Average age (range)	39 (22–75) years
*Education level*	
No school	3 (8.1)
Some primary	9 (24.3)
Completed primary	2 (5.4)
Some secondary	9 (24.3)
Completed secondary	14 (37.8)
*Has children*	37 (100)
*Average number of children (range)*	3.4 (1–9)
*Family planning method(s) ever used (multiple responses possible)*	
DMPA-SC (provider administered)	14 (37.8)
DMPA-SC (self-injection)	23 (62.2)
DMPA-IM	34 (94.4)^a^
Male condoms	25 (67.6)
Female condoms	4 (10.8)
Oral contraceptives	17 (45.9)
Implant	4 (10.8)
IUCD	2 (5.4)
Emergency contraception	7 (18.9)
Cycle beads	2 (5.4)
Other (e.g., natural, withdrawal, tubal ligation)	4 (10.8)
*Family planning method most recently used*	
DMPA-SC (provider administered)	1 (2.7)
DMPA-SC (self-injection)	18 (48.6)
DMPA-IM	9 (24.3)
Male condoms	2 (5.4)
Female condoms	0 (0)
Oral contraceptives	1 (2.7)
Implant	1 (2.7)
Intrauterine contraceptive device (IUCD)	0 (0)
Emergency contraception	0 (0)
Cycle beads	0 (0)
Other (e.g., natural, tubal ligation)	2 (5.4)

^a^One participant did not answer the question about past use of DMPA-IM

Out of the 64 family planning providers, 3 worked in private pharmacies in Blantyre and 3 worked in private clinics in Mangochi and Thyolo districts (data not shown). Just over half of providers were men (52%) and their average age was 38 years. Most providers had been offering family planning for more than 4 years and DMPA-SC (both provider administered and self-injection) for more than 1 year. On average, since beginning to offer DMPA-SC, each provider had counseled 531 clients on DMPA-SC self-injection (range 1–4000) and trained 198 clients to self-inject (range 0–1500).

In terms of female clients, the average age was 30 years and all except one had at least one child (data not shown). Most were married (77%) or had a sexual partner (20%), and for most (76%), their partner knew they were using family planning. The most commonly used method was DMPA-IM (77% had ever used).

### Men as family planning users

#### Men can and will offer participatory support

Participants discussed a variety of ways men had provided or could provide direct support to their partners to self-inject. As reported in the manuscript discussing the primary study objective [23], the main barrier to uptake of DMPA-SC self-injection was women’s fear of self-injecting. To address this concern, participants in 2 FGDs with men and 3 with providers discussed how men were attending or could attend counseling with their female partners to learn how to support her with the injection. For example, a participant from an FGD with men said:

*We come together to the facility and we got counseled together such that when one day she failed to open the tube, I helped her.*

Similarly, a participant from an FGD with providers said:

*I think if we can deal a lot with involving men to escort their wives whereby they can also get trained on how they can be injecting their wives, with this the wives may be encouraged to select self-injection.*

In men’s FGDs, participants whose partners had used DMPA-SC self-injection described helping to administer the actual injection when their wife got scared, helping their wife remember injection dates (e.g., by setting an alarm or marking the calendar), and assisting with DMPA-SC storage such as keeping the units out of children’s reach. For example, one participant said:

*Sometimes she is afraid to inject herself and I usually take the responsibility of injecting her. I also take the same responsibility in reminding her on the date she is supposed to reinject herself and I normally ask her if she can remember her day.*

Female clients were asked how their partners could support them if they decided to use DMPA-SC self-injection. Of the 30 female clients interviewed, 26 said that they would tell their partners if they decided to use DMPA-SC self-injection and 19 gave specific active ways their partner could or would support them. These included reminding them about when to re-inject and refilling the prescription when the units run out. About one-fifth of those who said they would tell their partner suggested that their partners could inject them. For example, one female client said:

*He can help in injecting me when I become afraid to inject myself.*

### Men as supportive partners

Some providers and female clients and most participants in the men’s FGDs felt that partner support would help women adopt and successfully use self-injection. Most male participants held personally favorable opinions toward DMPA-SC self-injection.

#### Why men are supportive

In FGDs with men, participants whose partners had self-injected described being supportive because they felt that self-injection saved their wives time which could be used to do other things for themselves and the household (e.g., chores), saved money due to reduced transportation costs, and limited the need to make multiple clinic trips due to crowded facilities, unavailability of providers, or stock outs. For example, one participant described:

*I see that it [self-injection] is a good thing as it alleviates the burden of movement and also it enables women to have ample time doing household chores, as it reduces time they spend in waiting for the service at the hospital.*

Participants in the men’s FGDs said they liked that self-injection allowed them to better know the whereabouts of their partners. Fewer clinic visits, including reduced transportation to and from the facility and time away from home, reduced their “doubt” about their wives, such as fear of infidelity. To illustrate this point, a men’s FGD participant remarked:

*Sometimes when they are coming here at hospital, one might think that she is not going to hospital but for a secret lover.*

Of the 26 female clients who said they would tell their partner if they decided to use DMPA-SC self-injection, 24 said they believed their partner would be supportive, or reassuring, of this decision, as described by this client:

*…as a schoolgirl there are a number of methods I follow to protect myself from unexpected pregnancy... So that method [DMPA-SC self-injection] is obvious that is used to protect from unwanted pregnancies hence he [my partner] will be in the forefront to make sure that I have completed my studies and if possible will get married and stay together.*

Echoing the sentiment that men like that self-injection helps women stay at home, one of the female clients said:

*Since most guys don’t like that women go to hospital for family planning, with privacy issues, he would prefer I self-inject here at home where it is convenient.*

#### How men are supportive

Participants in all the FGDs with men described men’s actual or potential role of offering emotional support to their partners to encourage uptake and continuation of DMPA-SC self-injection. Participants whose partners had used DMPA-SC self-injection said their support included telling jokes to reduce fear during injection, providing general encouragement to “be bold” and self-inject, embracing family planning use in general, and buying special foods to reassure their partner on injection day. For example, one participant said:

*What I liked most is that she told me that I should be reminding her if I feel like she will forget her reinjection dates, emphasizing that it is my duty to play a role in her use of DMPA-SC self-injection… I encouraged her to… make sure that if she feels that she will forget, she should hang the calendar on the wall and circle the reinjection dates clearly.*

Participants whose partners had not self-injected believed that if they encouraged their wives to use DMPA-SC self-injection, then women would not fear self-injecting and might be more interested in the practice, as this male participant described:

*We need just to encourage women that they should not be having fear because us men we have to support them when they want to self-inject. Therefore, this might make them to be bold to inject themselves.*

### Men as agents of change

#### Partner opposition or support affects women’s use of self-injection

In response to the questions about why women would or would not choose to self-inject, men, providers, and female clients stated that men play a distinct role in women’s use of DMPA-SC self-injection. About half of providers, one-fifth of clients, and some men discussed partner opposition as a barrier to DMPA-SC self-injection uptake and use. Providers and clients noted that covert users may not be willing to self-inject for fear that their partner would not be supportive if he found the units at home. For example, one female client said:

*For women who hide their use of contraceptives from their husbands because their husbands do not want them to be on it, self-injection might not be a good option for them, since they might feel that one day the injection units might bring disagreements in the family, which is different from provider administered where they just go to the facility, get injected, and come back without their husbands noticing about it.*

Male FGD participants discussed the issue of men’s opposition to DMPA-SC self-injection. In one FGD, they said this could deter women from trying it or force them into covert use. In another FGD, men acknowledged that by not openly supporting women, they were, in fact, inhibiting women from using DMPA-SC self-injection. For example, one male FGD participant said:

[*A*] *problem which women are facing is having fear to use this method of self-injecting at home because men are not supportive. Therefore, this makes women do it secretly.*

Participants in all men’s FGDs discussed that women would be interested in self-injection because it would benefit their lives, and they viewed women as being even more inclined to use DMPA-SC self-injection if their male partners and/or community leaders encouraged them to do so, as seen in the following discussion between 2 participants in a men’s FGD:

*Participant 1: Men are the reasons why women would opt for self-injection if we are to encourage them to do so since some of the women are afraid of injecting themselves. As men, we should make sure that we are in the forefront on encouraging women on the benefits of self-injection.*

*Participant 2: I feel that if these women are fed with proper and right information from their community leaders and service providers, they would be opting for this method.*

#### Men’s role in shifting norms

To increase men’s acceptability of self-injection, participants in one men’s FGD suggested that men whose wives self-inject should talk to other men about the practice. In another men’s FGD, participants suggested that male partners of self-injectors could disseminate messages to youth, with the aim of shifting norms for future generations. For example, one participant said:

*We have community youth clubs that move around in the communities disseminating various messages. Men are present in such gatherings, and I think that can be one of the platforms where we can introduce this method to them. Later, these men can carry these messages to their homes, and they would help to encourage women to opt for this method.*

Other participants from provider and men’s FGDs mentioned involving men in the development and dissemination of messaging in other fora, as well as making educational leaflets available in communities so that men could learn about DMPA-SC self-injection on their own time. Participants in all the men’s FGDs felt that women would be more inclined to self-inject if encouraged by male partners and/or community leaders. They suggested that expanding community involvement by educating village chiefs and husbands would work towards normalizing self-injection because men could help to change misconceptions in the community and promote an enabling environment. For example, one participant from a men’s FGD explained:

*If the HSAs would be invited in meetings which chiefs conduct with their subjects and give a talk on this section… In due course, men would be reminded frequently, hence they can be transformed with the passing of time.*

Similarly, providers urged community outreach and advocacy to encourage greater male involvement and subsequent uptake of DMPA-SC self-injection. More than half of providers were of the opinion that community involvement, particularly community leadership and male heads of household, would reinforce messaging around DMPA-SC self-injection. For instance, one provider said:

*As men are the head of the houses, we should involve them. We will not have problems. We should also do sensitization and health talks… Even the village heads should be told about DMPA-SC so that we can remove misconceptions in the villages.*

### Engaging men in social and behavioral change (SBC)

SBC efforts can play a role in increasing men’s support of contraceptive use through promotion of accurate knowledge of and favorable attitudes toward family planning and DMPA-SC. However, participants in all of the men’s FGDs reported that men do not receive enough messaging about DMPA-SC self-injection or family planning in general. One participant explained:

*The problem is that the women come on their own to collect the method without the men’s knowledge, leading to misunderstanding since men do have their own ideas. As such, men need to be involved.*

Most of the men’s FGD participants had positive views about DMPA-SC and self-injection. However, in all 4 men’s FGDs, participants also cited misconceptions, such as that women’s use of DMPA-SC affected men’s own physical and sexual health, including having reduced “sexual power.” For example, one participant stated:

*But the majority believe that when women, more especially my wife, when she takes this method of Sayana and it happens that I have slept with her, I do feel that something has entered in my body. Because when you went to bed, you do just fall asleep, and in my case… I don’t perform during sex as before. And even when I went to the farm [to work], I feel pain when I bend my waist.*

These myths were also reflected in provider and female client perceptions of how men viewed DMPA-SC, particularly, as one client described:

*Men feel that injectable contraceptive weakens their manhood power.*

Although providers and clients also noted that men ascribed to those myths about contraception in general and not exclusively DMPA-SC. One provider explained:

*For men, they think every family planning method weakens them. They say they do not perform in their homes sexually. They say they just do one round and they fall asleep.*

Providers also mentioned community-wide misconceptions, held by both men and women, such as DMPA-SC leading to female infertility, as this provider described:

*There were some rumors loitering around [the community] that when you inject yourself DMPA-SC on the stomach, the uterus gets burnt and you will never give birth.*

The majority of providers expressed the importance of engaging men in SBC in order to diminish misconceptions and increase user demand. They recommended engaging people in a number of ways, including community engagement with both men and women present, engaging men specifically, and counseling couples together. For example, one provider suggested:

*On misconceptions, we can encourage them, say they should be coming as couples when a wife wants to start taking the contraceptives, so that you should explain to both of them together.*

## Discussion

This is one of the first published analyses exploring the potential role of men in supporting DMPA-SC and self-injection use. We applied the male engagement framework in our interpretation of qualitative data from men, family planning providers, and female clients to inform future practices for male involvement in DMPA-SC and self-injection use. Specifically, we considered how men may be viewed as cooperative users, supportive partners, and agents of change even for this traditionally female-controlled method.

We suggest that self-injection has the potential to be both a female-controlled and a cooperative method, based on the ability for women to use it autonomously and the option to encourage male partner involvement. For the latter option, DMPA-SC self-injection could be considered a cooperative-method in cases where the female client welcomes engaging their partner in method use. Inclusion of this option of partners as potential co-users in training materials for providers and messaging for clients could, in turn, contribute to building more supportive environments to facilitate use of DMPA-SC self-injection.

It is critical to note that we do not suggest replacing the option for autonomous DMPA-SC self-injection. We recognize that there will always be covert users and women who do not want to involve their partners, and programs must continue to support such users. However, if programs expand their messaging on DMPA-SC self-injection to include men as potential co-users, they can enhance the use of self-injection for users who would like their partners to be involved. By engaging men as cooperative users, programs can shift gendered norms, such as the burden of pregnancy prevention falling on women [34].

We found that men in our study held favorable views about DMPA-SC self-injection and that they can and do support their partners to self-inject. However, an interesting theme that arose during our analysis was that many of the men’s FGD participants expressed reasons for liking self-injection that could be viewed as perpetuating gendered stereotypes and power imbalances. Men reported that self-injection gives women more time for household chores, allows men to be more involved in decision-making, and enables men to know their partners’ whereabouts. This perceived ability to increase trust by knowing their partner’s whereabouts may seem beneficial for the relationship at first glance, but keeping women under watch may limit their ability to move about freely outside of the home, which could ultimately impact their health [35, 36]. This is particularly a concern for women experiencing domestic violence, where accessing help may depend on gender-based violence screening by a provider [37–39]. Similarly, this could reduce women’s access to other essential services, including screening for sexually transmitted infections, HIV, and mental health issues which also require provider interaction. Though encouraging male participation in contraceptive counseling holds promise, there is a risk this could potentially lead to women’s autonomy being restricted. Recent studies of programs which promoted couples’ antenatal HIV testing documented that despite real benefits of these approaches, there were also unintended negative consequences such as clients attending services without a partner resulting in denial or delays in service-provision [40, 41].

Participants in this study reflected on how men’s support, or lack of support, affected women’s willingness to use DMPA-SC self-injection. If men support family planning and self-injection use, women will be inclined to use it; if men do not support family planning and self-injection use, women will be forced into covert usage or not use them at all. Previous research from Uganda and Malawi has also found that partner support of self-injection can enhance continuation and willingness to try the method [18, 27]. To address this, several projects have incorporated SBC messaging targeting men to build support for DMPA-SC self-injection use [42, 43]. Similar to our findings, these projects have determined that targeting messaging to both men and women can generate method demand.

The Family Planning High Impact Practice on Engaging Men and Boys in Family Planning advocates for assessing how gender norms affect male engagement because family planning investments can fall flat if programs fail to address gendered power dynamics [42]. We agree that conducting a gender analysis in a given social context as part of program design is critical to ensuring that men support women’s agency rather than act as gatekeepers and thwart women’s choice and autonomy. Related to this, men typically have higher social status than women in many contexts, therefore they have the potential to shift social norms by increasing the acceptability of DMPA-SC self-injection both at the interpersonal and community levels. Likewise, study participants voiced the importance of involving men at every stage of dissemination because of their influence on community norms and household decision-making. Recognizing that men often learn reproductive health information from their peers, some programs have taken on a peer-education framework whereby leaders and influential community members engage other men to effectively grow empathy around family planning and reproductive health [34]. We believe that engaging men, along with women and young people, in advocacy campaigns will encourage norms shifting at the community and global levels.

### Limitations

A number of limitations should be considered when interpreting our results. First, this analysis focused on answering a secondary objective of a study whose primary purpose was to develop and test a counseling message; therefore, the sampling approach may differ from a study whose sole purpose was to explore the potential role of men in supporting DMPA-SC self-injection. Our relatively small sample size and the fact that the data came from individuals in one city and 2 districts in Malawi may limit the generalizability of our findings. However, the same themes emerged in the data of different participant types (men, providers, and female clients) and thematic saturation was reached, strengthening our findings.

In addition, to meet the study’s primary objective of developing a counseling message, we intentionally sampled men who were familiar with DMPA-SC and self-injection, which could explain their highly favorable views of self-injection. It is possible that most men in these communities are not this accepting of self-injection. It is also possible that all participant types provided more favorable views on self-injection and positive roles men can play due to social desirability bias.

## Conclusions

Applying the male engagement framework to our qualitative data illuminated the roles men could play as cooperative users, supportive partners, and agents of change in DMPA-SC self-injection use and programming, and potentially for other contraceptive methods. While these data are from southern Malawi, we believe our findings may have broader applications, particularly for other southern African settings with similar sociocultural contexts, which should be explored through future research, implementation science, and practice. Given this, we propose shifting the view of men as a barrier to contraceptive use to considering men as a resource. This may allow us to harness the social capital of men and transform traditional power dynamics, therefore establishing more enabling environments to support autonomy and choice for DMPA-SC and self-injection use. Male engagement in family planning can be transformative and can lead to an enabling environment, as long as women and girls remain central to the conversation.

## Data Availability

The datasets used and/or analyzed during the current study are available from the corresponding author on reasonable request.

## Abbreviations

DMPA-SC: Subcutaneous depot medroxyprogesterone acetate
DMPA-IM: Intramuscular depot medroxyprogesterone acetate
FGD: Focus group discussion
HSA: Health surveillance assistant
MOH: Ministry of Health
SBC: Social and behavioral change
